# Successful lung-sparing resection of synchronous pleural mesothelioma and contralateral lung cancer

**DOI:** 10.1186/s40792-017-0336-9

**Published:** 2017-05-08

**Authors:** Naoko Imanishi, Yusuke Nabe, Masaru Takenaka, Yasuhiro Chikaishi, Koji Kuroda, Hirotsugu Noguchi, Kazuhiro Yatera, Fumihiro Tanaka

**Affiliations:** 1 Second Department of Surgery, University of Occupational and Environmental Health, Iseigaoka 1-1, Yahata-nishi-ku, Kitakyushu, 807-8555 Japan; 2 Department of Pathology and Cell Biology, School of Medicine, University of Occupational and Environmental Health, Kitakyushu, Japan; 3 Department of Respiratory Medicine, University of Occupational and Environmental Health, Kitakyushu, Japan

**Keywords:** Mesothelioma, Lung cancer, Synchronous, Lung-sparing surgery, Pleurectomy/decortication

## Abstract

**Background:**

Malignant pleural mesothelioma (MPM) is an uncommon malignant tumor, and its synchronous occurrence with primary lung cancer is extremely rare. Here, we report the first surgical case of synchronous MPM and contralateral lung adenocarcinoma. Extrapleural pneumonectomy (EPP) combined with surgery for contralateral lung cancer may not be tolerated, and a lung-sparing procedure including pleurectomy/decortication (P/D) can be an alternative to achieve complete resection.

**Case presentation:**

A 69-year-old male with right MPM and lung adenocarcinoma in the left upper lobe presented. Two lesions were judged to be synchronous MPM and lung cancer that were both potentially resectable clinical stage I diseases, and complete resection of both tumors was successfully achieved with right P/D following left upper division segmentectomy.

**Conclusions:**

P/D, not EPP, is a less invasive surgical procedure for MPM with curative intent and can be performed in combination with contralateral lung resection.

## Background

Malignant pleural mesothelioma (MPM) is an uncommon malignant tumor, and its synchronous occurrence with primary lung cancer is extremely rare [1–3]. A retrospective study of 3800 MPM patients identified only 18 patients (0.5%) with synchronous lung cancer [2]. Here, we report the first surgical case of synchronous MPM and contralateral lung adenocarcinoma, for which complete resection was achieved by right pleurectomy/decortication (P/D) following left upper division segmentectomy.

## Case presentation

**Fig. 1:**
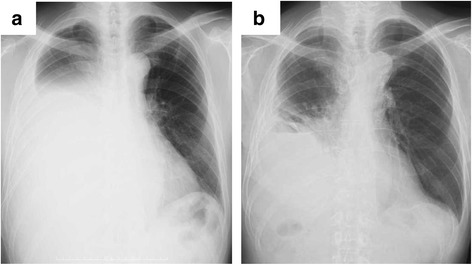
**a** Chest roentgenogram at presentation showing right pleural effusion. **b** Chest roentgenogram 2 months after right pleurectomy/decortication (P/D) following *left upper* division segmentectomy

**Fig. 2:**
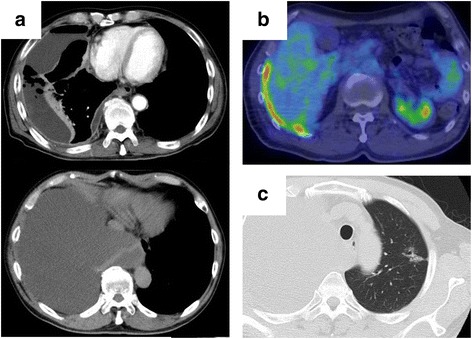
**a** Chest computed tomography (CT) at presentation showing *right* pleural effusion and focal thickening of the parietal pleura. **b** Positron emission tomography (PET) showing a moderate uptake of flouro-deoxy-glucose (FDG) with the maximum standardized uptake value of 6.6 in the thickened pleura. **c** Chest CT revealing a 2.5-cm “part-solid” ground-glass opacity (GGN) in the upper division of the *left* lung

A 69-year-old male with a history of occupational asbestos exposure presented with cough and shortness of breath, and chest roentgenogram showed the presence of right pleural effusion (Fig. 1). Chest computed tomography (CT) and positron emission tomography (PET) scan showed right pleural effusion and thickness of the parietal pleura with a moderate uptake of flouro-deoxy-glucose (FDG) (Fig. 2a, b), which was diagnosed as epithelioid MPM by thoracoscopic biopsy. At the same time, CT revealed a ground-glass nodule (GGN) in the left upper lobe (Fig. 2c), which was diagnosed as lung adenocarcinoma by trans-bronchial biopsy. Whole-body CT, PET scan, and brain magnetic resonance imaging revealed no nodal or distant metastasis, and the two lesions were judged to be synchronous MPM and lung cancer that were both potentially resectable clinical stage I diseases.

Pulmonary function test showed that a forced vital capacity (FVC) and expiratory volume in 1 s (FEV_1.0_) before surgery were 3.52 L (1.91 L/mm^2^) and 2.44 L (1.43 L/mm^2^), respectively. Lung ventilation/perfusion scan showed decreased scan uptake in the right side (ventilation, 32% in the right and 68% in the left; perfusion, 35% in the right and 68% in the left, respectively). Based on the ventilation scan data, the predicted FEV_1.0_ after right EPP combined was only 0.96 L/mm^2^, indicating that the patient could not tolerate right EPP combined with left upper lobe resection.

**Fig. 3:**
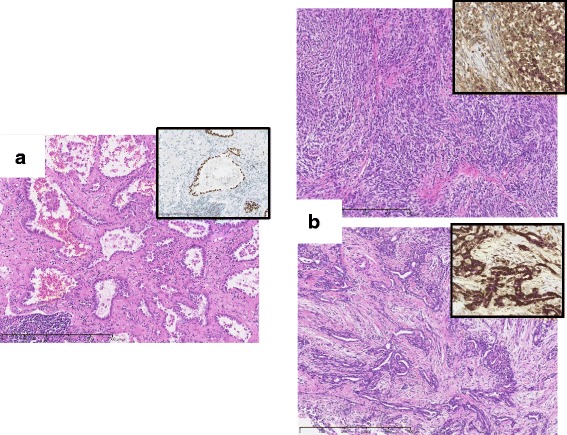
**a** Pathological features of *left* lung tumor showing papillary growth of atypical tumor cells that were positive for thyroid transcription factor-1 (TTF-1). **b** Pathological features of *right* pleural tumor showing a combination of sarcomatoid (*upper*) and epithelioid (*lower*) components. Tumor cells of both components were positive for calretinin

Accordingly, we decided to perform a lung-sparing surgery for each tumor. First, we performed left upper division segmentectomy through video-assisted thoracic surgery (VATS), and then performed operation for right MPM after 3-weeks interval from the first operation. Macroscopic complete resection was achieved with extended P/D with combined resection of involved diaphragm and pericardium. The pathological diagnosis was synchronous biphasic MPM (pathologic stage III, due to tumor invasion into the diaphragm and chest wall) and papillary adenocarcinoma of the lung (pathologic T1bN0M0, stage IA2) (Fig. 3). The patient had an uneventful recovery, and adjuvant chemotherapy with cisplatin plus pemetrexed has been prescribed.

Here, we presented the first case of synchronous MPM and contralateral lung cancer that were successfully removed with lung-sparing surgical procedures. The aim of surgery with curative intent for MPM is to obtain macroscopic resection, but which surgical procedure, EPP or P/D, is more beneficial remains controversial [1, 4]. EPP, a very radical procedure, might provide more survival advantage as compared with a lung-sparing P/D, but its superiority over P/D has been proved in some studies but not in others. A recent meta-analysis comparing EPP and P/D showed that EPP was associated with higher perioperative mortality (4.5 versus 1.7%) but provided no better postoperative survival [5]. Based on these results, more surgeons have recently tended to prefer P/D [6], although the choice depends on clinical factors and on individual surgical judgment and expertise [1, 4].

In the present case, the predicted postoperative FEV_1.0_ after right EPP alone, right EPP plus left upper division segmentectomy, and right EPP plus left upper lobectomy were 0.96, 0.70, and 0.43 L/mm^2^, respectively, indicating that the patient may marginally tolerate right EPP alone and may not tolerate right EPP combined with left lung resection. For such case, systemic chemotherapy may be commonly employed, but P/D, not EPP, in combination with left lung resection may be an alternative choice of treatment. In the present case, we actually achieved complete resection of both tumors by maintaining good performance status by lung-sparing surgical procedures (right P/D and left upper division segmentectomy).

With respect to treatment sequence, we did not perform P/D first. P/D may be sometimes associated with prolonged air-leak postoperatively, which may lead to delayed treatment for lung cancer. Accordingly, we first conducted segmentectomy for lung cancer. With respect to the treatment modality for lung cancer, surgery may be a better choice for the cure as compared with radiation therapy including stereotactic radiotherapy (SRT). In addition, radiation therapy may be sometimes associated with pneumonitis, which may lead to delay of subsequent treatment. Partial resection may be appropriate only for pure GGN, not for part-solid tumor, as a curative-intent surgery.

## Conclusions

P/D can be performed for patients who might not be eligible for EPP and was actually performed even in combination with contralateral lung resection.

## Abbreviations

CT: Computed tomography
FDG: Flouro-deoxy-glucose
FEV_1.0_: Forced expiratory volume in 1 s
GGN: Ground-glass nodule
MPM: Malignant pleural mesothelioma
P/D: Pleurectomy/decortication
PET: Positron emission tomography
VATS: Video-assisted thoracic surgery
